# Retraction note: Histopathological and clinical evaluation of Kombucha tea and Nitrofurazone on cutaneous full-thickness wounds healing in rats: an experimental study

**DOI:** 10.1186/s13000-016-0566-3

**Published:** 2016-11-02

**Authors:** Fardin Barati, Javad Javanbakht, Farajollah Adib-Hashemi, Ehsan Hosseini, Reyhaneh Safaeie, Mojtaba Rajabian, Mostafa Razmjoo, Reza Sedaghat, Mehdi Aghamohammad Hassan

**Affiliations:** 1 Department of Clinical Science, Faculty of Veterinary Medicine, Tehran University, Tehran, Iran; 2 Department of Pathology, Faculty of Veterinary Medicine, Tehran University, Tehran, Iran; 3 Faculty of Para Veterinary Medicine, Ilam University, Ilam, Iran; 4 Graduate, Faculty of Veterinary Medicine, Tehran University, Tehran, Iran; 5 Food Hygiene Department, University of Shahekord, Shahekord, Iran; 6 Faculty of Veterinary Medicine, Razi University, Kermanshah, Iran; 7 Department of Pathology, Faculty of Medicine Science, Shahed University, Tehran, Iran

## Retraction

The Editor-in-Chief and Publisher have retracted this article [1] because the scientific integrity of the content cannot be guaranteed. An investigation by the Publisher found it to be one of a group of articles we have identified as showing evidence suggestive of attempts to subvert the peer review and publication system to inappropriately obtain or allocate authorship. This article showed evidence of plagiarism (most notably from the articles cited [2–4]), peer review and authorship manipulation.
